# An Innovation Perspective to Explore the Ecology and Social Welfare Efficiencies of Countries

**DOI:** 10.3390/ijerph19095113

**Published:** 2022-04-22

**Authors:** Z-John Liu, Minh-Hieu Le, Wen-Min Lu

**Affiliations:** 1 Department of Business Administration, Ling Tung University, No. 1, Ling Tung Rd., Taichung 408213, Taiwan; liuzjohn@gmail.com; 2 Faculty of Business Administration, Ton Duc Thang University, No. 19 Nguyen Huu Tho Street, Tan Phong Ward, District 7, Ho Chi Minh City 700000, Vietnam; leminhhieu@tdtu.edu.vn; 3 Department of International Business Administration, Chinese Culture University, No. 55, Hwa-Kang Road, Shilin District, Taipei 114, Taiwan

**Keywords:** data envelopment analysis, network-based approach, directional distance function, ecology efficiency, social welfare efficiency

## Abstract

This study aims to measure the ability of 29 countries in producing competitive products and services that fulfill individual needs and improve the level of welfare with less utilization of natural resources. We build a two-stage network production process model to investigate the ecology efficiency and social welfare efficiency of the countries and then further discriminate the efficient countries in post-analysis. The two-stage network directional distance function is applied to assess the efficiencies of countries, and the network-based ranking approach is used to further discriminate the efficient countries following the panel data between the years 2013 and 2016. Results show that Poland and Spain are strongly referenced by other countries in the ecology stage, whereas Bulgaria, the United States, and Sweden are leaders in the social welfare stage. A remarkable observation is an absence of countries’ efficiency in both ecology and social welfare efficiencies. Most of the 29 countries have lower efficiency in the social welfare stage than in the ecology stage. This study suggests the strengths and highlights the weaknesses of the countries to help the governments efficiently improve and operate their countries.

## 1. Introduction

Over the last few decades, we have seen a participatory tendency in both environmental governance and knowledge production [1]. Environmental awareness is an essential component of both public and private decision-making [2]. Capturing the most economic gains while utilizing the fewest resources and resulting in the least damage to the environment is a critical issue for social development [3]. As society becomes ever more developed, units from different levels, that is, human beings, companies, and government, all are starting to pay attention to the importance of the environment and social welfare. Many cities throughout the world have set climate change mitigation targets, but activities to implement these targets have proven ineffective thus far. There may be confusion about who is accountable for acting, how to connect with a diverse variety of stakeholders, how to define goals, and how to measure performance [4]. Recently, Jones, Donaldson [5] vigorously encourage researchers related to management to consider social welfare in their empirical research. The idea of ecology efficiency and social welfare efficiency offers a comprehensive view for policymakers and government to achieve better national performance with the sustainable development goal [3]. The study of ecology efficiency has been previously performed on a national scale [6,7,8,9]. Moraes, Wanke [10] have recently studied social welfare and labor efficiency at a regional level. Remarkably, Lefebvre, Perelman [11] assess the overall welfare state performance of the 28 European Union countries based on eight-year (2005–2012) period data. Although efficiency measurement in the public sector is traditionally long, and there is an immense number of researchers who publish the results of productivity comparisons of countries, it is not easy to identify and correctly evaluate the outcomes [11]. Balancing ecology efficiency and social welfare efficiency can better attain equilibrium and sustainable development [12]. Whereas ecology efficiency refers to the ability of countries to produce goods and services with less effect on the environment and lower levels of natural resources consumption [13], social welfare efficiency refers to poverty reduction and inequality alleviation, and protection against disease, unemployment, and ignorance [11].

Management performance evaluation is a difficult task because it involves multiple inputs and outputs [14]. Designing, evaluating, and monitoring activities, programs, and policies aimed at improving countries’ growth at both the national and international levels is a difficult process that necessitates the use of a range of instruments. The requirement to measure economic, social, and environmental dimensions adds to the complexity of progress assessment [15]. Measurements of ecology efficiency and social welfare efficiency have been performed by many previous authors using the ratio approach, stochastic frontier analysis (SFA), or data envelopment analysis (DEA) [3,6,7,11,12,16,17,18,19]. Robaina-Alves, Moutinho [16] measured the environmental and resource efficiency of European countries by using data from two separate periods that can perceive the difference in the efficiency level before and after the achievement of the Kyoto protocol in 2005. Robaina-Alves, Moutinho [16] used the stochastic frontier approach in their study. However, DEA seems to be the most widely applied method because of the advantages of processing multiple inputs and outputs. Moreover, the previous studies measured the efficiency of countries without considering and analyzing the intermediate products and linking activities [8,12,13]. Unlike traditional DEA, which treats a system as a “black box,” network DEA considers its underlying structure to get more insightful conclusions [20]. Quality development is not the objective pursued by economic development, but an instrument to accomplish sustainable economic and social development [12]. A multi-stage DEA model that links the ecology efficiency and social welfare efficiency to measure the overall efficiency of a country is suggested, as the overall efficiency can be obtained only when all subsequent processes work well [21,22]. For the conventional DEA model, if decision-making units (DMUs) are simultaneously effective, no differentiation exists for efficient leaders [23]. As noted in [16], a suggestion for future research is to uncover factors that are the reasons for efficient or inefficient countries. To further measure and explore the merits of efficient leaders, previous authors have applied different ranking methods including the super-efficiency DEA model [12], cross efficiency evaluation method [24,25], TOPSIS technique [26], rough set approach [27], and network-based ranking approach [28,29]. Especially, Liu, Lu [30] have suggested a network-based ranking approach as a useful and powerful efficiency ranking tool to distinguish the benchmark and highlight the strengths and weaknesses of DMUs (Liu et al. 2009).

Perceived from the current literature review, this study aims to measure the capacity of the countries to produce competitive products and services that satisfy individual needs and improve the level of well-being with less use of natural resources. We explore the ecology efficiency and social welfare efficiency of countries as two subsequent processes of a network production process structure to determine the best nation for benchmarking by applying a directional distance function (DDF) based model for efficiency measurement in two-stage network DEA. Inefficient countries may learn from pioneers to improve their efficiency. In addition, this study combines a network-based ranking approach [28,29,30] to further distinguish the benchmark countries. At a macro level for the countries, the findings are of great relevance to help policymakers set policies and plan budgets to implement these policies and achieve better performance. In summary, the current study contributes to the related literature review as follows:

First, a novel network production process framework in two-stage network DEA is produced for measuring the ecology efficiency and social welfare efficiency of countries by using DDF based model with consideration of undesirable outputs.

Second, this study is the first to use a network-based approach, which is a unique and powerful method, to further discriminate the benchmark countries in the context of ecology efficiency and social welfare efficiency. The results suggest the strengths and highlight the weaknesses of the countries that help the government efficiently improve and operate their countries.

## 2. Literature Review

Climate change is one of the most difficult issues confronting the globe today, and it is critical to have effective policies in place to handle its consequences [31,32]. Countries in the world are seriously dealing with the challenges and pressures from creating waste and pollution by many firms [9]. The governments need to consider integrating the economic, environmental, and social dimensions in their policy-making process to reach sustainable development, which requires minimizing the environmental concerns and maximizing economic and social indicators [9,33]. Economic efficiency together with environmental efficiency create ecological efficiency [7].

Tena Medialdea, Prieto Ruiz [34] recognized the requirement for ecological studies that address the role of humans as ecosystem members. Ecological efficiency (abbreviated eco-efficiency) has aroused increasing attention from the government, practitioners, and scholars in recent years [3,6,9,16,35]. Schaltegger and Sturm [36] proposed the concept of “eco-efficiency” as “a business link to sustainable development,” and the World Business Council revealed the term in 1992 as the index of economic and environmental efficiency, namely as a management strategy that links financial and environmental performance to create more value with less ecological impact [37]. According to Dyckhoff and Allen [38], the best-known definition of eco-efficiency is from World Business Council for Sustainable Development (WBCSD) “Eco-efficiency is achieved by the delivery of competitively priced goods and services that satisfy human needs and bring the quality of life, while progressively reducing ecological impact and resource intensity throughout the life-cycle to a level at least in line with the Earth’s estimated carrying capacity ”. Zhou, Ang [39] provide a non-radial DDF approach to evaluate the energy and CO_2_ performance of electricity production by using data in 2005 from 126 countries. In terms of CO_2_ performance, OECD countries surpassed non-OECD countries, and OECD countries were equivalent to non-OECD countries in terms of energy performance [39]. Robaina-Alves, Moutinho [16] measured the eco-efficiency of European countries by applying the stochastic frontier approach using data in two separate periods including before (2000–2004) and after (2005–2011) the Kyoto Protocol. The efficiency levels of European countries between two periods before and after the creation of environmental targets are compared in the study [16]. Liu and Liu [40] measured the low carbon economy efficiency with a three-stage model to compare the largest 20 CO_2_ emitting nations from 2000 to 2012. First, they applied DEA, using energy consumption, capital stock, and labor force as input factors, and GDP and CO_2_ emissions as (undesirable) output factors, to get efficiency for each nation and compute the slack at the input and output, then applied SFA to remove the influence of external environmental variables on the slack. Finally, they recalculated the efficiency using updated input and output components to reflect the government’s ability to establish a low-carbon economy. According to their results, during the studied period, the performance was getting worse in these low carbon economies. Wu, Yin [41] used a two-stage DEA model to assess environmental efficiency for China’s 30 provinces and eight regions, with the production subsystem as the first stage and the pollution treatment subsystem as the second. Interestingly, both of the papers included undesirable outputs in their models. The recently published article that is related to ecology efficiency of Yang and Zhang [6] suggested an extended DEA approach, which incorporates global benchmark technology, DDF, and a bootstrapping method to explore the dynamic trends of Chinese regional eco-efficiency in the 2003–2014 period. Pais-Magalhães, Moutinho [17] applied the DEA approach to measure the eco-efficiency of 15 European countries by using data in the 2001–2015 period. The countries, including Belgium, Luxembourg, Sweden, the Netherlands, and the United Kingdom, show better ecology performance in comparison with the other European countries.

The connection between ecology and human social welfare have gained visibility in the past few years [5,10,12,17]. It is important to put emphasis on human welfare at the social level and integrate social and economic objectives in the research [42]. However, the context of social welfare is rather complex. The satisfaction of basic and secondary needs experienced by individuals in a community is referred to as social welfare [43]. Social welfare is a normative term that various persons or social groups use to reflect on the ends—the “greater good”—that public policy should pursue to better society’s status quo. Importantly, when it comes to many issues of public policy, people mean different things based on their self- and other-regarding preferences, as well as socio-demographic variables such as education, income, wealth, and influence [44]. As noted in the research work of Hall, Giovannini [45], the ecosystem is equally important as the human well-being system, as the resources and services of human activities are provided by the ecosystem. Nissi and Sarra [46] based their research work on Hall, Giovannini [45], and address the measure of well-being in the context of Italian urban areas using an integrated DEA-entropy approach. Their findings show significant dualism between northern and southern cities, revealing significant variations in many facets of human and ecological well-being. Lefebvre, Perelman [11] provide a definition and a technique to evaluate the efficiency of the public sector. The authors then measure the efficiency of European welfare countries and their development over time by applying the DEA approach. Wang and Feng [12] used super-efficiency DEA and Malmquist index approach to measure the ecology welfare efficiency of China in the 2006–2018 period. Recently, Moraes, Wanke [10] reveal the endogeneity between labor efficiency and social welfare by applying a two-stage network DEA approach using data from 2013 to 2016 in Brazil.

## 3. Research Design

### 3.1. Two-Stage Production Process of Countries

**Figure 1:**
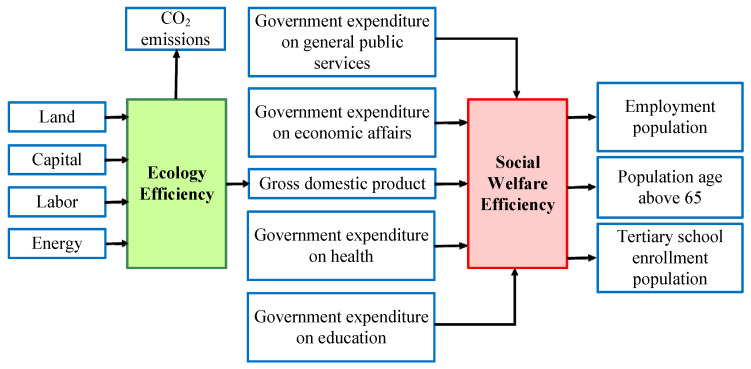
Two-stage production process of countries.

This article studies the ecology efficiency and social welfare efficiency of 29 countries in the 2013–2016 period. The data were collected from British Petroleum (BP), International Monetary Fund (IMF), Organization for Economic Cooperation and Development (OECD), and World Bank (WB). These databases are commonly used sources for research. Details are shown in Table 1. The selection of input, intermediate, and output variables is based on the related research listed in the social science citation index (SSCI). The initial selection of the variables is explained as follows. For the first stage, ecology efficiency, a nation requires land, capital, and labor and will consume energy to generate gross domestic product (GDP) and undesirable gas emissions (i.e., CO_2_). For the second stage, social welfare efficiency, government expenditure on general public services, economic affairs, health, and education along with the first stage output, GDP, as intermediate to generate outputs including employment population, population age above 65, and tertiary school enrollment population. Figure 1 shows the two different stages to examine the internal structure, namely, ecology efficiency and social welfare efficiency stages. The operational definition of each of the variables is shown in Table 1.

Table 2 present the descriptive statistics for the variables of 29 countries. The variables have a positive connection (Table 3), which followed the isotonic condition employed to determine the efficient level. Table 2 indicates that most of the variables have a non-normal distribution (Kolmogorov-Smirnov test significant). This finding shows that using the DEA technique is the right option because the method requires no assumption of normality for data [47].

### 3.2. Research Method

This article uses the multivariate evaluation approach that simultaneously measures various dimensions of countries’ efficiency to overcome the single-dimension shortcoming of the traditional approach. This article uses the two-stage network DDF in evaluating the internal network production structures to understand the countries’ ecology and social welfare efficiencies [13,35]. To examine the merits of each country under different circumstances, this article incorporates multiple DEA specifications and a social network approach to determine the strengths and weaknesses of the countries [30]. The linear programming issues are shown below.

Let us consider a set of n countries (k=1,…,m). For a decision-making unit k, m inputs $x_{ak}(a=1,\ldots ,m)$ are used to produce $z_{bk}(b=1,\ldots ,l)$, intermediate outputs in the first stage, and then $z_{bk}$ plus a new set of factors $z_{ck}$(c=1,…,g) produce h outputs in the second stage $\left(y_{dk},d=1,\ldots ,h\right)$.

Assume that the set of production possibilities for both inputs and outcomes is convex. The DDF two-stage network is defined as follows:

(1) $$\begin{array}{l} DDF\left(x,z,y;g_{x},g_{y}\right)\\ =Max\left\{\delta +\beta :\left(x-\delta g_{x},z,y+\beta g_{y}\right)\in T\left(x,z,y\right)\right\}. \end{array}$$

The following is a definition of the technology set:

T(x,z,y):$x_{ak}$ can produce the intermediate outputs $z_{bk}$ in the first process; $z_{bk}$ and $z_{ck}$ can produce the final $y_{dk}$ in the second process.

According to Fried, Lovell [48], the direction vector $g=\left(g_{x},g_{y}\right)$ should be chosen by the researcher before evaluating the DDF. In this paper, we consider the direction to be $g=\left(g_{x}=x,g_{y}=y\right)$. As a result, the following linear programs can describe the inefficiency measure of the target country of the technology set under convex constraints:

(2) $$\begin{array}{l} {\vec{DDF}}^{}=Max\;\delta_{o}^{}+\beta_{o}^{}\\ \sum_{k=1}^{n_{}}\lambda_{ko}^{}x_{ak}^{}\leq x_{ao}-\delta_{o}^{}g_{aox},\;\;a=1,\ldots ,m,\\ \sum_{k=1}^{n}\lambda_{ko}^{}z_{bk}^{}\geq z_{bo},\;\;b=1,\ldots ,l,\\ \sum_{k=1}^{n}\mu_{ko}^{}z_{bk}^{}\leq z_{bo},\;\;b=1,\ldots ,l,\\ \sum_{k=1}^{n_{}}\mu_{ko}^{}z_{ck}^{}\leq z_{co},\;\;c=1,\ldots ,g,\\ \sum_{k=1}^{n}\mu_{ko}^{}y_{dk}^{}\geq y_{do}+\beta_{o}^{}g_{doy},\;\;d=1,\ldots ,h,\\ \sum_{k=1}^{n}\lambda_{ko}^{}=1,\\ \sum_{k=1}^{n}\mu_{ko}^{}=1,\\ \lambda_{k}^{},\mu_{k}^{}\geq 0, \end{array}$$

where $\lambda_{ko}^{}$ and $\mu_{ko}^{}$ are the intensity variables corresponding to the first and second processes for a given country. The best solution $\lambda_{ko}^{*}$ for an observed country demonstrates if a country k serves as a role model for the observed country in the first stage. The optimal solution $\mu_{ko}^{*}$ is the same definition in the second stage. As a result, the first stage’s production efficiency, $EE_{o}^{}=1-\delta_{o}^{}$, which is ecology efficiency. Ecology efficiency ranges between 0 and 1. The efficiency of the second stage in these sets is defined as $SE_{o}^{}=1/\left(1+\beta_{o}^{}\right)$, which is the social welfare efficiency. The social welfare efficiency is between 0 and 1. These variables indicate that the target country is efficient in the first and second stages if the $EE_{o}^{}$ and $SE_{o}^{}$ are equal to unity.

The concept of the reference-share measure is introduced below. With high probability, many DEA specifications are used in the efficiency evaluation. Using a variety of DEA specifications allows for examining the merits of each DMU under different situations, thus laying the foundation for further differentiation. For any DEA specification t, the linear programming problem (2) is represented as follows:

(3) $$\begin{array}{l} {\vec{DDF}}^{t}=Max\;\delta_{o}^{t}+\beta_{o}^{t}\\ \sum_{k=1}^{n_{}}\lambda_{ko}^{t}x_{ak}^{t}\leq x_{ao}-\delta_{o}^{t}g_{aox},\;\;a=1,\ldots ,m,\\ \sum_{k=1}^{n}\lambda_{ko}^{t}z_{bk}^{t}\geq z_{bo},\;\;b=1,\ldots ,l,\\ \sum_{k=1}^{n}\mu_{ko}^{t}z_{bk}^{t}\leq z_{bo},\;\;b=1,\ldots ,l,\\ \sum_{k=1}^{n_{}}\mu_{ko}^{t}z_{ck}^{t}\leq z_{co},\;\;c=1,\ldots ,g,\\ \sum_{k=1}^{n}\mu_{ko}^{t}y_{dk}^{t}\geq y_{do}+\beta_{o}^{t}g_{doy},\;\;d=1,\ldots ,h,\\ \sum_{k=1}^{n}\lambda_{ko}^{t}=1,\\ \sum_{k=1}^{n}\mu_{ko}^{t}=1,\\ \lambda_{k}^{t},\mu_{k}^{t}\geq 0, \end{array}$$

Each specification t can be thought of as a competition game round. As a result, the initial DEA issue has been expanded from a one-round competition to a multi-round competition as a result of this action. Because the efficiency score is tied in the first round of this competition, extra game rounds may be requested to allow each DMU to demonstrate its worth in a variety of conditions. The champion is then determined based on the cumulative results. The efficiency calculation accounts for all conceivable input/output combinations.

The values of $\lambda_{ko}^{t*}$ and $\mu_{ko}^{t*}$ denote the optimal solution in Model 3. In the DEA setting, small efficient countries with lower input/output levels are likely to achieve higher $\lambda_{ko}^{t*}$ and $\mu_{ko}^{t*}$ than large efficient countries. Normalizing the $\lambda_{ko}^{t*}$ and $\mu_{ko}^{t*}$ could remove the effect of country size and render the approach applicable to both the constant and variable returns to scale models.

Let $E_{t}$ be the index set for the observed country’s reference set. Under DEA specification t, the contribution of the kth country’s ath input to the oth country in the reference set is specified as

(4) $$Ix_{ako}^{t}=\lambda_{ko}^{t*}x_{ak}^{t}/\sum_{k\in E_{t}}^{}\lambda_{ko}^{t*}x_{ak}^{t},\;0<Ix_{ako}^{t}\leq 1,a=1,\ldots ,m.$$

Similarly, with DEA specification t, the contribution of the kth country’s bth intermediate to the oth country in the reference set is defined as

(5) $$MIz_{bko}^{t}=\frac{1}{2}\left(\lambda_{ko}^{t*}z_{bk}^{t}/\sum_{k\in E_{t}}^{}\lambda_{ko}^{t*}z_{bk}^{t}\right)+\frac{1}{2}\left(\mu_{ko}^{t*}z_{bk}^{t}/\sum_{k\in E_{t}}^{}\mu_{ko}^{t*}z_{bk}^{t}\right),\;0<MIz_{bko}^{t}\leq 1,b=1,\ldots ,l.$$

Under DEA specification t, the contribution of the kth country’s cth additional input to the oth country in the reference set is specified as

(6) $$Iz_{cko}^{t}=\mu_{ko}^{t*}z_{ck}^{t}/\sum_{k\in E_{t}}^{}\mu_{ko}^{t*}z_{ck}^{t},\;0<Iz_{cko}^{t}\leq 1,\;c=1,\ldots ,g.$$

Under DEA specification t, the contribution of the kth country’s dth output to the oth country in the reference set is defined as

(7) $$Oy_{dko}^{t}=\mu_{ko}^{t*}y_{dk}^{t}/\sum_{k\in E_{t}}^{}\mu_{ko}^{t*}y_{dk}^{t},0<Oy_{dko}^{t}\leq 1,\;d=1,\ldots ,h.$$

The total of the input and output components of a normalized reference weight is obtained by averaging them out.

(8) $$IMO1_{ko}^{t}=\frac{1}{m+l}{\left[\sum_{a=1}^{m}Ix_{ako}^{t}+\sum_{b=1}^{l}MIz_{bko}^{t}\right]}_{}$$

(9) $$IMO2_{ko}^{t}=\frac{1}{l+g+h}{\left[\sum_{b=1}^{l}MIz_{bko}^{t}+\sum_{c=1}^{g}Iz_{cko}^{t}+\sum_{d=1}^{h}Oy_{dko}^{t}\right]}_{}$$

(10) $$A1={\left[\sum_{t=1}^{T}IMO1_{ko}^{t}\right]}_{}\;\mathrm{and}\;A2={\left[\sum_{t=1}^{T}IMO2_{ko}^{t}\right]}_{}$$

The value $T=\left(2^{m}-1\right)\left(2^{l}-1\right)\left(2^{g}-1\right)\left(2^{h}-1\right)$ is the number of combinations tested by the DEA model, whereas A1 and A2 are square matrices of size n×n. Matrix elements A1 and A2 represent the combined power of the oth unit supporting the kth unit or the cumulative effect of the oth unit endorsing the kth unit.

We observe that A1 and A2 can be viewed as adjacency matrices of a directed and weighted network, where nodes are DMUs, and links express the amount of endorsement from one unit to the other. Bonacich and Lloyd [49] proposed alpha-centrality, an eigenvector-like metric, to distinguish the significance of nodes in a directed network. The significance of each node is embedded in the following formulation’s solutions I1 and I2:

(11) I1=αA1⋅I1+e and I2=αA2⋅I2+e

where e is a unit vector and α is an arbitrary constant indicating the relevance of endogenous versus exogenous influences. Each vector element, $I1_{k}$ and $I2_{k}$, provides the scores used to distinguish the efficient units in the first and second stages, respectively.

The efficient units for each I/M/O factor can also be differentiated. When Formula (10) is rearranged, the result is

(12) $$A1=\frac{1}{m+l}{\left[\sum_{a=1}^{m}A1I_{a}^{}+\sum_{b=1}^{l}A1M_{b}^{}\right]}_{}$$

(13) $$A2=\frac{1}{l+g+h}{\left[\sum_{b=1}^{l}A2M_{b}^{}+\sum_{c=1}^{g}A2I_{c}^{}+\sum_{d=1}^{h}A2O_{d}^{}\right]}_{}$$

(14) $$\begin{array}{l} A1I_{a}^{}={\left[\sum_{t=1}^{T}Ix_{ako}^{t}\right]}_{},\;a=1,\ldots ,m;\\ A1M_{b}^{}={\left[\sum_{t=1}^{T}MIz_{bko}^{t}\right]}_{},\;b=1,\ldots ,l;\\ A2M_{b}^{}={\left[\sum_{t=1}^{T}MIz_{bko}^{t}\right]}_{},\;b=1,\ldots ,l;\\ A2I_{c}^{}={\left[\sum_{t=1}^{T}Iz_{cko}^{t}\right]}_{},\;c=1,\ldots ,g;\\ A2O_{d}^{}={\left[\sum_{t=1}^{T}Oy_{dko}^{t}\right]}_{},\;d=1,\ldots ,h. \end{array}$$

It is worth noting that $A1I_{a}$, $A1M_{b}$, $A2I_{c}$, $A2O_{d}$ are square matrices of order n. Given that $A1M_{b}$ and $A2M_{b}$ are the aggregated reference matrices for the same intermediate factors in the first and second stages, the actual contribution of each intermediate component should be averaged. One can define

(15) $$AM_{b}=\frac{1}{2}\left(A1M_{b}+A2M_{b}\right)=\left[\sum_{t=1}^{T}MIz_{bko}^{t}\right].$$

The matrices $A1I_{a}$, $AM_{b}$, $A2I_{c}$, $A2O_{d}$ are thus the reference matrices for each I/M/O. Each matrix member indicates the aggregated endorsement of an observed unit to the kth unit in the reference set via a specific I/M/O factor. When the alpha centrality notion is applied to these matrices, the following results are obtained:

(16) $$\begin{array}{l} I1I_{a}=\alpha A1I_{a}\cdot I1I_{a}+e\\ IM_{b}=\alpha AM_{b}\cdot IM_{b}+e\\ I2I_{c}=\alpha A2I_{c}\cdot I2I_{c}+e\\ I2O_{d}=\alpha A2O_{d}\cdot I2O_{d}+e \end{array}$$

where the column vectors $I1I_{a}$, $IM_{b}$, $I2I_{c}$ and $I2O_{d}$ hold the centrality scores of each unit since each I/M/O factor is regarded as the standard for that specific factor among all units.

Internally, the unit strength of these I/M/O factors can also be compared. The sum of each row element of the matrices $A1I_{a}$, $AM_{b}$, $A2I_{c}$, $A2O_{d}$ indicates the overall endorsement a unit obtains from its peers as a result of the contribution of a specific I/M/O factor. As a result, the endorsement from all other units’ overall specifications to an efficient unit k via a specified I/M/O factor w equals

(17) $$IMOS_{k}^{w}=\left\{ \begin{array}{l} \sum_{o=1}^{n}\sum_{t=1}^{T}Ix_{ako}^{t},\;a=1,\ldots ,m\;for\;w=1,\ldots ,m,\;and\\ \sum_{o=1}^{n}\sum_{t=1}^{T}MIz_{bko}^{t},\;b=1,\ldots ,l\;for\;w=m+1,\ldots ,m+l,\;and\\ \sum_{o=1}^{n}\sum_{t=1}^{T}Iz_{cko}^{t},\;c=1,\ldots ,g\;for\;w=m+l+1,\ldots ,m+l+g,\;and\\ \sum_{o=1}^{n}\sum_{t=1}^{w}Oy_{dko}^{t},\;d=1,\ldots ,h\;for\;w=m+l+g+1,\ldots ,m+l+g+h. \end{array}\right.$$

where w is a combined I/M/O factor index in this case. For an efficient unit k, the higher the $IMOS_{k}^{w}$ larger the contribution of the wth factor to the unit’s efficiency. To simplify comparison, the relative intensity of an I/M/O factor w defined in formula (17) is magnified using the formula:

(18) $$IMO_{k}^{w}=\frac{{\left(IMOS_{k}^{w}\right)}^{2}}{\sum_{w=1}^{m+l+g+h}{\left(IMOS_{k}^{w}\right)}^{2}}.$$

As a result, $IMO_{k}^{w}$ denotes the relative strength of an I/M/O factor w among all factors within an efficient unit k.

## 4. Empirical Analysis

### 4.1. Ecology Efficiency and Social Welfare Efficiency for Countries

Initially, this study conducts a preliminary analysis of the ecology efficiency and social welfare efficiency for countries by running on full specifications including inputs/intermediate/undesirable output/additional inputs/outputs. Table 4 shows the efficiencies of each country at each stage.

There is a total of six efficient countries at the ecology efficiency stage (Czech Republic, New Zealand, Poland, Spain, Switzerland, and Turkey) and four at the social welfare efficiency stage (Bulgaria, Italy, Sweden, United States). No country is efficient at both stages, and 19 countries are inefficient in two stages. As shown in Table 4, the average values of efficiency scores are 0.8315 and 0.4949 for ecology efficiency and social welfare efficiency, respectively, which emphasize a potential improvement of the social welfare efficiency for the countries. These preliminary results present an overview of the efficiencies of each country, but further differentiation is required to determine the best performer.

### 4.2. Analysis of Benchmarking of Production Factors

**Figure 2:**
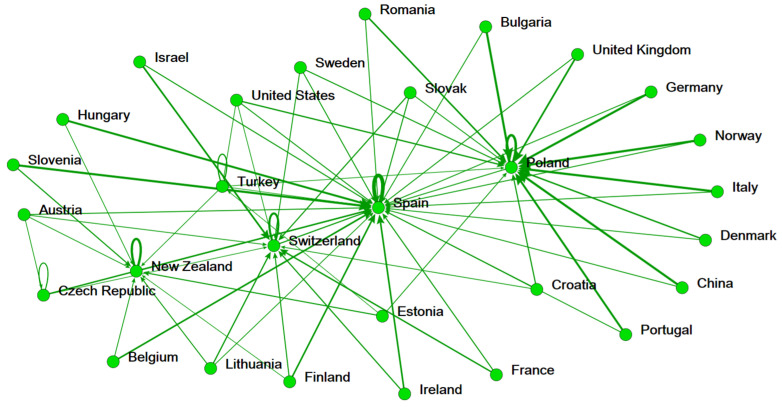
Reference network of 29 nations in the ecology efficiency stage.

**Figure 3:**
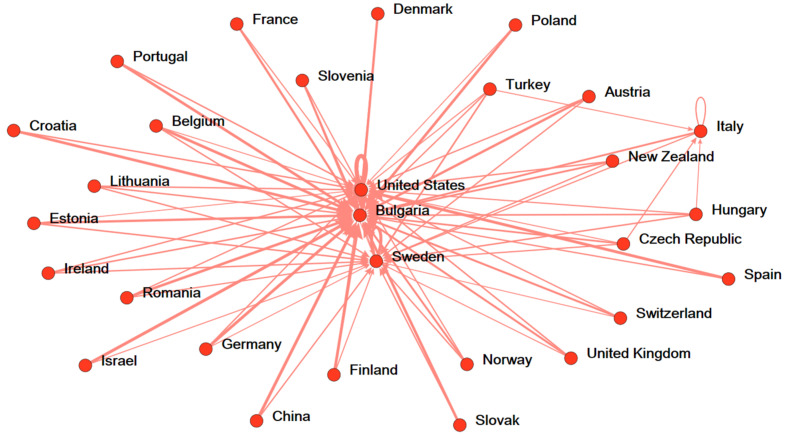
Reference network of 29 nations in social welfare efficiency.

**Figure 4:**
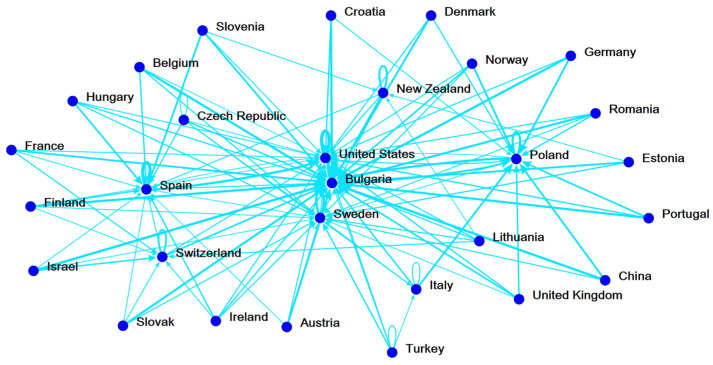
Reference network of 29 nations in overall efficiency.

Next, this study used a network-based ranking approach to discover the most efficient country in each stage and each factor (inputs, intermediate, undesirable output, additional inputs, and outputs). The strengths of each country are also confirmed. Figure 2, Figure 3 and Figure 4 show the analysis results are aggregated from a total of 1575 DEA runs. In Figure 2, the ecology efficiency of countries is visibly presented by the accrued reference networks. The endorsing connections are identified by the thickness and the darkness of the lines in the figure. Typically, if a country delegated by a node in the figure has more lines approach, its ranking is higher. Poland and Spain (Bulgaria, United States, and Sweden) are strongly referenced by other countries in the ecology stage (social welfare stage) (Figure 2 and Figure 3). Our findings are different from the findings of [16], which considers the ecology efficiency in two distinct periods (2000–2004 and 2005–2011). However, the switch in the position of countries in terms of ecology efficiency due to the considered period in our study is different from the study of Robaina-Alves, Moutinho [16]. Our study is conducted using data when Kyoto Protocol was adopted for a second commitment period, whereas the study of Robaina-Alves, Moutinho [16] was performed using data in periods of the first commitment (2005–2011) and before Kyoto Protocol entered into force (2000–2004). These findings show that there is an evolution of the ecology efficiency ranking of some European countries among periods. One example of such awareness is the adoption of the Kyoto Protocol. Kutlu [50] demonstrated that the Kyoto Protocol’s adoption and implementation aided the environment by reducing GHG emissions relative to (real) GDP. Therefore, it can be explained that the Kyoto Protocol helped in improving the ecology efficiency of the European countries [50]. Similarly, top benchmarks for overall efficiency are assigned to Bulgaria, the United States, and Sweden (Figure 4).

Furthermore, Table 5, Table 6, Table 7 and Table 8 reveal the calculated strength indices of input/undesirable output/intermediate/additional inputs/output factors for each country. In terms of input (land, capital, labor, and energy) and undesirable (CO_2_) factors at the ecology stage, 14 countries in Europe (Bulgaria, Croatia, Czech Republic, Estonia, Finland, Hungary, Ireland, Israel, Lithuania, New Zealand, Romania, Slovenia, Spain, Slovak Republic, and Austria) and Israel are in the top list (Table 5 and Table 6).

In terms of energy and CO_2_ factors, our study confirms the results of the authors [39] in which China and the United States are found to have low performance. In their research [39], the authors explained the relatively poor electric power generation efficiency and the coal-dominated fuel input in electric power generation of these large countries [39]. Our results show that the inefficient countries in energy and CO_2_ factors have enormous potential to decrease consumption of energy and CO_2_ emission. Finland, New Zealand, and Spain are the best performers in the intermediate factor of GDP (Table 6).

At the social welfare stage, nine countries, including Belgium, Finland, France, New Zealand, Norway, Poland, Spain, Sweden, and the United Kingdom are the leaders in the input and output factors of government expenditure on general public services, government expenditure on economic affairs, government expenditure on health, government expenditure on education and population age above 65, employment population, tertiary school enrollment population, respectively (Table 7 and Table 8).

## 5. Conclusions

Ecology efficiency and social welfare efficiency improvement are the the most important policy options for countries. This study aims to measure the ability of the 29 countries in producing competitive products and services that fulfill individual needs and improve the level of welfare with less utilization of natural resources. This study builds a two-stage network production process model to investigate the ecology efficiency and social welfare efficiency of the countries.

At the preliminary analysis of efficiencies, efficiency scores obtained from the two-stage network DDF function show six efficient countries at the ecology stage and four efficient countries at the social welfare stage. No country is efficient at both stages. The findings also demonstrate that most of the 29 countries have lower efficiency in the social welfare stage than in the ecology stage. The empirical results provide policymakers with a better awareness of the ecology efficiency and social welfare efficiency of the countries.

Furthermore, we examine efficient countries and confirm leading countries for learners. The findings suggest the strengths and highlight the weaknesses of the countries in terms of input/undesirable output/intermediate/additional input/output factors that assist the governments to improve and operate their country efficiently. It may be argued that nations producing large unfavorable outputs may not function eco-efficiently and hence have a great possibility to save the maximum amount of energy. Furthermore, nations with low energy consumption may be more eco-efficient and have a lower capacity to minimize undesired outputs. For example, countries such as Norway, Switzerland, China, the United States, Italy, and the United Kingdom have considerable opportunities for reducing energy usage and CO_2_ emissions. Norway, the United States, Lithuania, Turkey, and China still have enormous potential to increase GDP. At the social welfare stage, countries including Turkey, Lithuania, China, Bulgaria, and Denmark have room to improve their social welfare efficiency by reducing the quantity of government expenditure on general public services, government expenditure on economic affairs, government expenditure on health, government expenditure on education while increasing population age above 65, employment population, and tertiary school enrollment population. From a macro perspective, both the ecology efficiency and social welfare efficiency determine a country’s overall efficiency level. According to the empirical findings, policy makers’ varying degrees of interest preference affect the ecology efficiency and social welfare efficiency.

Following the limitations of this study, we suggest helpful guidance for future research. First, this study takes the data from various sources, although cross-database is our contribution in this study, our sample focuses on countries from different regions. Future research may consider studying the countries in the same region. Second, although this study uses panel data to investigate the ecology efficiency and social welfare efficiency of the countries, this does not allow us to compare the efficiency levels of countries in different distinct periods. Future research may consider dividing into distinctive periods (i.e., before the Kyoto Protocol entered into force period, the first commitment of the Kyoto Protocol period, and the second commitment of the Kyoto Protocol period).

## Figures and Tables

**Table 1:** Definitions of variables.

Variables	Definitions	Units	Sources
***Inputs for stage 1***			
Land	Land area is the overall area of a country, excluding inland water bodies, national claims to the continental shelf, and exclusive economic zones. In most situations, significant rivers and lakes are included in the concept of inland water bodies.	Square kilometer	WB
Capital	The cost of new fixed assets plus the net change in inventories.	Million USD	WB and IMF
Labor	All groupings of people aged 15 and up who fit the International Labor Organization’s (ILO) definition of economically active population.	People	WB and IMF
Energy consumption	The total amount of recycled and non-renewable energy consumed.	Million tons	BP
***Intermediate***			
GDP	A measure of a country’s economic position, the market price of all final goods and services produced in the country during the year.	Million USD	WB and IMF
***Output for stage 1***			
CO_2_ emission (undesirable)	Greenhouse gases emitted by the combustion of fossil fuels.	Million tons	BP
***Additional input for stage 2***			
Government expenditure on general public services	Government spending on executive and legislative bodies, financial and fiscal affairs, external affairs, public debt transactions, general services, foreign economic aid, R&D, basic research, general public services, and transfers of a general nature between different levels of government.	Million USD	IMF
Government expenditure on economic affairs	Government spending covers general economic, commercial, and labor affairs, agriculture, forestry, fishing and hunting, fuel and energy, mining, manufacturing and construction, transportation, communication, other industries, R&D economic affairs, and economic affairs.	Million USD	IMF
Government expenditure on health	Medical products, appliances, and equipment, outpatient services, hospital services, public health services, R&D health, and health are all examples of government spending.	Million USD	IMF
Government expenditure on education	Total general (local, regional, and national) government education spending (current, capital, and transfers), expressed as a percentage of GDP. It includes government spending funded by transfers from international sources.	Million USD	IMF
***Outputs for stage 2***			
Employment population	The employment to population ratio denotes the percentage of a country’s population that is employed. Employment is defined as persons of working age who were engaged in any activity to produce goods or provide services for pay or profit during a short reference period, whether at work during the reference period (i.e., who worked in a job for at least one hour) or not at work due to temporary absence from a job or working-time arrangements. Working-age people are generally considered to be those aged 15 and up.	People	WB
Population age above 65	A country’s population aged 65 and up. The population is calculated using the de facto definition, which includes all residents regardless of legal status or citizenship.	People	WB
Tertiary school enrollment population	Total population of higher school students, regardless of age.	People	OECD

Note: WB is World Bank; OECD is Organization for Economic Co-operation and Development; IMF is International Monetary Fund; BP is British Petroleum.

**Table 2:** Descriptive statistics of input/intermediate/output factors for DEA analysis.

Factors	Units	Mean	Minimum	Maximum	Std.Dev.	K-S Test ^a^
Land	Square kilometer	827,475.00	20,141.10	9,388,211.00	2,345,481.00	*p* < 0.01
Capital	Million USD	423,543.00	6289.40	4,866,509.00	1,077,108.00	*p* < 0.01
Labor	People	42,108,745.00	684,412.80	786,639,089.00	146,348,397.00	*p* < 0.01
Energy	Million tons	152.00	2.90	1709.00	406.00	*p* < 0.01
GDP	Million USD	1,622,478.00	24,316.70	17,707,452.00	3,713,023.00	*p* < 0.01
CO_2_ emission	Million tons	581.00	13.30	7864.00	1698.00	*p* < 0.01
Government expenditure on general public services	Million USD	84,903.00	975.40	1,016,089.00	193,448.00	*p* < 0.01
Government expenditure on economic affairs	Million USD	76,921.00	1127.90	862,939.00	187,640.00	*p* < 0.01
Government expenditure on health	Million USD	106,239.00	1272.90	1,594,631.00	295,824.00	*p* < 0.01
Government expenditure on education	Million USD	82,605.00	1439.80	1,088,363.00	209,487.00	*p* < 0.01
Employment population	People	48,204,755.00	755,646.10	911,642,187.00	169,726,118.00	*p* < 0.01
Population age above 65	People	9,559,414.00	246,012.60	130,420,422.00	24,963,669.00	*p* < 0.01
Tertiary school enrollment population	People	2,784,949.00	51,473.60	37,472,107.00	7,595,033.00	*p* < 0.01

Note: ^a^ Kolmogorov–Smirnov test.

**Table 3:** Correlation coefficients for input/intermediate/output factors.

Factors	X1	X2	X3	X4	Z1	UEY1	EX1	EX2	EX3	EX4	Y1	Y2	Y3
X1	1.000												
X2	0.979 **	1.000											
X3	0.826 **	0.900 **	1.000										
X4	0.993 **	0.994 **	0.858 **	1.000									
Z1	0.938 **	0.907 **	0.635 **	0.937 **	1.000								
UEY1	0.977 **	0.996 **	0.921 **	0.990 **	0.879 **	1.000							
EX1	0.813 **	0.753 **	0.395 *	0.804 **	0.959 **	0.712 **	1.000						
EX2	0.972 **	0.999 **	0.908 **	0.990 **	0.896 **	0.993 **	0.740 **	1.000					
EX3	0.741 **	0.657 **	0.264	0.719 **	0.912 **	0.614 **	0.986 **	0.639 **	1.000				
EX4	0.874 **	0.812 **	0.479 **	0.859 **	0.980 **	0.779 **	0.989 **	0.798 **	0.972 **	1.000			
Y1	0.813 **	0.889 **	0.999 **	0.846 **	0.615 **	0.912 **	0.372 *	0.898 **	0.240	0.457 *	1.000		
Y2	0.885 **	0.952 **	0.985 **	0.919 **	0.744 **	0.962 **	0.534 **	0.959 **	0.406 *	0.601 **	0.980 **	1.000	
Y3	0.945 **	0.978 **	0.956 **	0.961 **	0.815 **	0.986 **	0.624 **	0.978 **	0.513 **	0.696 **	0.948 **	0.980 **	1.000

Notes: **, * correlations are significant at level 0.05, 0.01, respectively. X1 is land; X2 is capital; X3 is labor; X4 is energy; Z1 is GDP; UEY1 is CO_2_ emission; EX1 is government expenditure on general public services; EX2 is government expenditure on economic affairs; EX3 is government expenditure on health; EX4 is government expenditure on education; Y1 is employment population; Y2 is population age above 65; Y3 is tertiary school enrollment population.

**Table 4:** Ecology efficiency and social welfare efficiency for each country.

Countries	Ecology Efficiency	Ranking	Social Welfare Efficiency	Ranking
Austria	0.8491	(15)	0.2003	(27)
Belgium	0.9716	(9)	0.1926	(28)
Bulgaria	0.736	(22)	1	(1)
China	0.7932	(20)	0.6329	(8)
Croatia	0.6216	(26)	0.4554	(14)
Czech Republic	1	(1)	0.2758	(22)
Denmark	0.6241	(25)	0.6452	(7)
Estonia	0.9097	(13)	0.2443	(23)
Finland	0.8522	(14)	0.2109	(25)
France	0.9263	(10)	0.4044	(16)
Germany	0.7105	(23)	0.5329	(11)
Hungary	0.9235	(11)	0.2875	(21)
Ireland	0.9983	(7)	0.5633	(9)
Israel	0.9828	(8)	0.3779	(18)
Italy	0.8019	(19)	1	(1)
Lithuania	0.8026	(18)	0.5199	(12)
New Zealand	1	(1)	0.1426	(29)
Norway	0.7708	(21)	0.7555	(6)
Poland	1	(1)	0.5056	(13)
Portugal	0.6168	(27)	0.9165	(5)
Romania	0.8332	(17)	0.3724	(19)
Slovak	0.835	(16)	0.3994	(17)
Slovenia	0.9191	(12)	0.2438	(24)
Spain	1	(1)	0.2046	(26)
Sweden	0.573	(28)	1	(1)
Switzerland	1	(1)	0.421	(15)
Turkey	1	(1)	0.3035	(20)
United Kingdom	0.7053	(24)	0.5448	(10)
United States	0.3555	(29)	1	(1)
Average	0.8315		0.4949	
Total efficient countries	6		4	

**Table 5:** Rankings of a nation’s sensitivity to each input factor at the ecology stage.

Input Factors											
Land			Capital			Labor			Energy		
Country	Eigenvector Centrality		Country	Eigenvector Centrality		Country	Eigenvector Centrality		Country	Eigenvector Centrality	
Bulgaria	0.223316	(1)	Bulgaria	0.223575	(1)	Bulgaria	0.223195	(1)	Bulgaria	0.223435	(1)
Croatia	0.223316	(1)	Croatia	0.223575	(1)	Croatia	0.223195	(1)	Croatia	0.223435	(1)
Czech Republic	0.223316	(1)	Czech Republic	0.223575	(1)	Czech Republic	0.223195	(1)	Czech Republic	0.223435	(1)
Estonia	0.223316	(1)	Estonia	0.223575	(1)	Estonia	0.223195	(1)	Estonia	0.223435	(1)
Finland	0.223316	(1)	Finland	0.223575	(1)	Finland	0.223195	(1)	Finland	0.223435	(1)
Hungary	0.223316	(1)	Hungary	0.223575	(1)	Hungary	0.223195	(1)	Hungary	0.223435	(1)
Ireland	0.223316	(1)	Ireland	0.223575	(1)	Ireland	0.223195	(1)	Ireland	0.223435	(1)
Israel	0.223316	(1)	Israel	0.223575	(1)	Israel	0.223195	(1)	Israel	0.223435	(1)
Lithuania	0.223316	(1)	Lithuania	0.223575	(1)	Lithuania	0.223195	(1)	Lithuania	0.223435	(1)
New Zealand	0.223316	(1)	New Zealand	0.223575	(1)	New Zealand	0.223195	(1)	New Zealand	0.223435	(1)
Romania	0.223316	(1)	Romania	0.223575	(1)	Romania	0.223195	(1)	Romania	0.223435	(1)
Slovenia	0.223316	(1)	Slovenia	0.223575	(1)	Slovenia	0.223195	(1)	Slovenia	0.223435	(1)
Spain	0.223316	(1)	Spain	0.223575	(1)	Spain	0.223195	(1)	Spain	0.223435	(1)
Slovak Republic	0.223316	(1)	Slovak Republic	0.223575	(1)	Slovak Republic	0.223195	(1)	Slovak Republic	0.223435	(1)
Austria	0.205360	(15)	Austria	0.205614	(15)	Austria	0.205250	(15)	Austria	0.205524	(15)

Notes: As there are 29 nations in our sample, this table presents only the top 15 nations. Numbers in parentheses are nations’ ranks for input factors at the ecology stage.

**Table 6:** Rankings of a nation’s sensitivity to each intermediate/undesirable output factor at the ecology stage.

Intermediate			Undesirable Output		
GDP			CO_2_ Emissions		
Country	Eigenvector Centrality		Country	Eigenvector Centrality	
Finland	0.257666	(1)	Bulgaria	0.223299	(1)
New Zealand	0.257666	(1)	Croatia	0.223299	(1)
Spain	0.257666	(1)	Czech Republic	0.223299	(1)
Romania	0.254200	(4)	Estonia	0.223299	(1)
Estonia	0.250589	(5)	Finland	0.223299	(1)
Czech Republic	0.248091	(6)	Hungary	0.223299	(1)
Slovak Republic	0.247188	(7)	Ireland	0.223299	(1)
Ireland	0.247185	(8)	Israel	0.223299	(1)
Hungary	0.236101	(9)	Lithuania	0.223299	(1)
Austria	0.234099	(10)	New Zealand	0.223299	(1)
Belgium	0.229228	(11)	Romania	0.223299	(1)
Slovenia	0.219672	(12)	Slovenia	0.223299	(1)
Germany	0.198977	(13)	Spain	0.223299	(1)
Poland	0.196221	(14)	Slovak Republic	0.223299	(1)
Sweden	0.189684	(15)	Austria	0.205371	(15)

Notes: As there are 29 nations in our sample, this table presents only the top 15 nations. Numbers in parentheses are nations’ ranks for intermediate/undesirable output factors at the ecology stage.

**Table 7:** Rankings of a nation’s sensitivity to each additional input factor at the social welfare stage.

Input Factors											
Government Expenditure on General Public Services			Government Expenditure on Economic Affairs			Government Expenditure on Health			Government Expenditure on Education		
Country	Eigenvector Centrality		Country	Eigenvector Centrality		Country	Eigenvector Centrality		Country	Eigenvector Centrality	
Belgium	0.218450	(1)	Belgium	0.218398	(1)	Belgium	0.218170	(1)	Belgium	0.218552	(1)
Finland	0.218450	(1)	Finland	0.218398	(1)	Finland	0.218170	(1)	Finland	0.218552	(1)
France	0.218450	(1)	France	0.218398	(1)	France	0.218170	(1)	France	0.218552	(1)
New Zealand	0.218450	(1)	New Zealand	0.218398	(1)	New Zealand	0.218170	(1)	New Zealand	0.218552	(1)
Norway	0.218450	(1)	Norway	0.218398	(1)	Norway	0.218170	(1)	Norway	0.218552	(1)
Poland	0.218450	(1)	Poland	0.218398	(1)	Poland	0.218170	(1)	Poland	0.218552	(1)
Spain	0.218450	(1)	Spain	0.218398	(1)	Spain	0.218170	(1)	Spain	0.218552	(1)
Sweden	0.218450	(1)	Sweden	0.218398	(1)	Sweden	0.218170	(1)	Sweden	0.218552	(1)
United Kingdom	0.218450	(1)	United Kingdom	0.218398	(1)	United Kingdom	0.218170	(1)	United Kingdom	0.218552	(1)
Romania	0.216727	(10)	Romania	0.215053	(10)	Romania	0.217085	(10)	Romania	0.216275	(10)
Switzerland	0.216690	(11)	Switzerland	0.215020	(11)	Switzerland	0.217062	(11)	Switzerland	0.216250	(11)
Slovak Republic	0.210317	(12)	Slovak Republic	0.210327	(12)	Slovak Republic	0.210022	(12)	Slovak Republic	0.210432	(12)
Ireland	0.210312	(13)	Ireland	0.210324	(13)	Ireland	0.210017	(13)	Ireland	0.210430	(13)
Austria	0.209197	(14)	Austria	0.209141	(14)	Austria	0.208929	(14)	Austria	0.209291	(14)
Estonia	0.200968	(15)	Estonia	0.201529	(15)	Estonia	0.200584	(15)	Estonia	0.200968	(15)

Notes: As there are 29 nations in our sample, this table presents only the top 15 nations. Numbers in parentheses are nations’ ranks for additional input factors at the social welfare stage.

**Table 8:** Rankings of a nation’s sensitivity to each output factor at the social welfare stage.

Output Factors								
Employment Population			Population Age above 65			Tertiary School Enrollment Population		
Country	Eigenvector Centrality		Country	Eigenvector Centrality		Country	Eigenvector Centrality	
Belgium	0.218366	(1)	Belgium	0.217690	(1)	Belgium	0.218349	(1)
Finland	0.218366	(1)	Finland	0.217690	(1)	Finland	0.218349	(1)
France	0.218366	(1)	France	0.217690	(1)	France	0.218349	(1)
New Zealand	0.218366	(1)	New Zealand	0.217690	(1)	New Zealand	0.218349	(1)
Norway	0.218366	(1)	Norway	0.217690	(1)	Norway	0.218349	(1)
Poland	0.218366	(1)	Poland	0.217690	(1)	Poland	0.218349	(1)
Spain	0.218366	(1)	Spain	0.217690	(1)	Spain	0.218349	(1)
Sweden	0.218366	(1)	Sweden	0.217690	(1)	Sweden	0.218349	(1)
United Kingdom	0.218366	(1)	United Kingdom	0.217690	(1)	United Kingdom	0.218349	(1)
Romania	0.214503	(10)	Romania	0.215933	(10)	Romania	0.216508	(10)
Switzerland	0.214475	(11)	Switzerland	0.215902	(11)	Switzerland	0.216489	(11)
Slovak Republic	0.210314	(12)	Slovak Republic	0.209581	(12)	Slovak Republic	0.210222	(12)
Ireland	0.210312	(13)	Ireland	0.209579	(13)	Ireland	0.210220	(13)
Austria	0.209108	(14)	Austria	0.208465	(14)	Austria	0.209100	(14)
Estonia	0.201508	(15)	Estonia	0.200674	(15)	Estonia	0.201343	(15)

Notes: As there are 29 nations in our sample, this table presents only the top 15 nations. Numbers in parentheses are nations’ ranks for output factors at the social welfare stage.

## Data Availability

The data that support the findings of this study are available from the corresponding author, upon reasonable request.
